# A Comprehensive Characterization of Monoallelic Expression During Hematopoiesis and Leukemogenesis *via* Single-Cell RNA-Sequencing

**DOI:** 10.3389/fcell.2021.702897

**Published:** 2021-10-13

**Authors:** Ruiqing Fu, Pengfei Qin, Xianghui Zou, Zhangli Hu, Ni Hong, Yun Wang, Wenfei Jin

**Affiliations:** ^1^Shenzhen Key Laboratory of Microbiology and Gene Engineering, College of Life Sciences and Oceanography, Shenzhen University, Shenzhen, China; ^2^Key Laboratory of Optoelectronic Devices and Systems of Ministry of Education and Guangdong Province, College of Optoelectronic Engineering, Shenzhen University, Shenzhen, China; ^3^School of Food Engineering and Biotechnology, Hanshan Normal University, Chaozhou, China; ^4^Shenzhen Key Laboratory of Gene Regulation and Systems Biology, School of Life Sciences, Southern University of Science and Technology, Shenzhen, China

**Keywords:** single-cell RNA sequencing, constitutive monoallelic expression, random monoallelic expression, bone marrow mononuclear cells, leukemia

## Abstract

Single-cell RNA-sequencing (scRNA-seq) is becoming a powerful tool to investigate monoallelic expression (MAE) in various developmental and pathological processes. However, our knowledge of MAE during hematopoiesis and leukemogenesis is limited. In this study, we conducted a systematic interrogation of MAEs in bone marrow mononuclear cells (BMMCs) at single-cell resolution to construct a MAE atlas of BMMCs. We identified 1,020 constitutive MAEs in BMMCs, which included imprinted genes such as *MEG8*, *NAP1L5*, and *IRAIN*. We classified the BMMCs into six cell types and identified 74 cell type specific MAEs including *MTSS1*, *MOB1A*, and *TCF12*. We further identified 114 random MAEs (rMAEs) at single-cell level, with 78.1% single-allele rMAE and 21.9% biallelic mosaic rMAE. Many MAEs identified in BMMCs have not been reported and are potentially hematopoietic specific, supporting MAEs are functional relevance. Comparison of BMMC samples from a leukemia patient with multiple clinical stages showed the fractions of constitutive MAE were correlated with fractions of leukemia cells in BMMCs. Further separation of the BMMCs into leukemia cells and normal cells showed that leukemia cells have much higher constitutive MAE and rMAEs than normal cells. We identified the leukemia cell-specific MAEs and relapsed leukemia cell-specific MAEs, which were enriched in immune-related functions. These results indicate MAE is prevalent and is an important gene regulation mechanism during hematopoiesis and leukemogenesis. As the first systematical interrogation of constitutive MAEs, cell type specific MAEs, and rMAEs during hematopoiesis and leukemogenesis, the study significantly increased our knowledge about the features and functions of MAEs.

## Introduction

Mammalian genomes including human genome are diploid, with one haploid inherited from mother and the other inherited from father. Although it is usually assumed that genes are expressed from both alleles of the diploid genome, some genes are expressed from only one allele, which is called monoallelic expression (MAE) (Eckersley-Maslin and Spector, 2014a; Reinius and Sandberg, 2015; Chess, 2016; Han et al., 2020). One kind of the most studied MAE is genomic imprinting, in which either the paternal or the maternal allele of imprinted genes is expressed. The parental-origin-specific MAEs of imprinted genes have been demonstrated to play an important role in embryonic development (Reik and Walter, 2001; Ferguson-Smith, 2011). However, the constitutive MAEs such as genomic imprinting only account for a small fraction of total MAEs. Random MAEs (rMAEs), that stochastically determine one allele to be transcribed and lead to different cells of the organism expressing different alleles, are much prevalent (Gimelbrant et al., 2007; Deng et al., 2014; Reinius and Sandberg, 2015; Chess, 2016). The earliest reported rMAE was random X-chromosome inactivation that was described >60 years ago (Lyon, 1961). X-chromosome inactivation mainly balances X-chromosome gene dosages between male and female, which carry one and two copies of X-chromosome, respectively (Lyon, 1986). In contrast to chromosome-wide rMAE caused by random X-chromosome inactivation, autosomal rMAE on immunoglobulins and odorant receptors has been well studied in the past decades (Pernis et al., 1965; Hozumi and Tonegawa, 1976; Chess et al., 1994). A lot of autosomal rMAEs interspersing over the genome was detected in recent decade (Gimelbrant et al., 2007; Deng et al., 2014; Reinius and Sandberg, 2015). Recent studies showed that a considerable proportion of the rMAE should be attributed to RNA transcriptional bursting, which describes the switching kinetics of the two alleles expressing periodically (Kim and Marioni, 2013; Choi et al., 2019; Larsson et al., 2019; Ochiai et al., 2020). However, the genome-wide landscape of autosomal rMAE in hematopoiesis is largely unexplored.

Single-cell RNA-sequencing (scRNA-seq) provides a unique opportunity to analyze rMAE genome wide (Gimelbrant et al., 2007; Deng et al., 2014; Borel et al., 2015). Tools have been designed to perform rMAE analysis originally for full-length scRNA-seq data, simultaneously dealing with the transcriptional bursting, e.g., SCALE (Jiang et al., 2017) and scBase (Choi et al., 2019), but they are not suitable for 3′-scRNA-seq data. Analyses of rMAE in different cell lineages/types suggest that rMAE is established during development (Eckersley-Maslin et al., 2014b; Gendrel et al., 2014). However, the reported fractions of autosomal rMAE in human genome are quite different from study to study, ranging from 5 to 76.4% (Gimelbrant et al., 2007; Deng et al., 2014; Borel et al., 2015; Kim et al., 2015; Reinius et al., 2016). The contradictions between these studies may be caused by different cell lineages/types and false positives of rMAE identifications in these studies. Several studies have explored the relationships between MAE and tumor (Meehan et al., 2007; Walker et al., 2012; Polson et al., 2013; Al Seraihi et al., 2018; Silcock et al., 2019). For example, MAE of *TP53* was observed in mutated brain tumors while not in healthy tissues, indicating MAE potentially is associated with tumor progression (Walker et al., 2012). However, these studies only analyzed a very limited number of cells and did not conduct systematic analysis on MAE. In order to systematically characterize the MAEs during hematopoiesis and leukemogenesis, we identified and analyzed the constitutive MAEs, cell type specific MAEs, and rMAEs using large scale scRNA-seq data.

## Materials and Methods

### Sample Information

The sample information and scRNA-seq data have been described in our recent study (Qin et al., 2021). In short, bone marrow mononuclear cells (BMMCs) were collected from a boy diagnosed with acute lymphoblastic leukemia (ALL) separately at four clinical time points, i.e., diagnosis, refractory, complete remission, and relapse. In addition, the whole-genome sequencing (WGS) data were generated from the boy’s saliva sample and BMMC samples from the four time points, except the complete remission stage (Zhang et al., 2018).

### Identification of Genomic Single-Nucleotide Variant and Filtering

Reads from WGS data were trimmed using cutadapt (Martin, 2011), and then mapped to the hg38 human reference genome with BWA (Li and Durbin, 2010). We used CNVnator (Abyzov et al., 2011) to call copy number variations (CNVs) in each of the samples, with default parameters. GATK best practice pipeline (McKenna et al., 2010; DePristo et al., 2011) was applied to process the duplicate-marked raw reads to analysis-ready mapped reads. HaplotypeCaller mode of the GATK was performed for each of the samples and then joint calling was conducted across the samples. Low-quality (QUAL ≤ 30) single nucleotide variants (SNVs) were removed and only autosomal bi-allelic SNVs were kept. To avoid the *cis*-influence from CNVs, we removed the SNVs located in the detected CNV regions for each sample. We also removed the SNVs that were not in dbSNP (v147). Finally, we removed the putative somatic mutations. According to the empirical data, a SNV was identified as a somatic mutation if its UMI count and percentage of the alternative-allele (alt-allele) were not larger than 10 and less than 40%, respectively.

### Single-Cell RNA-Sequencing Data Process and Cell-Type Inference

The scRNA-seq raw data were processed following 10X Genomics workflow, using Cell Ranger (suite 2), with hg38 human reference genome. The basic transcriptomic analyses have been described in our recent study (Qin et al., 2021), namely, filtering cells, inferring major cell types, and identifying the cell states (i.e., normal cells or leukemia cells) in BMMCs.

The identified SNVs in WGS data were examined in mapped reads of scRNA-seq data, as well as the information of cell barcode and UMI in matched reads. Thus, it yielded the allelic UMI counts for each given SNV for each cell. The variant allele frequency (VAF) of alt-allele can be estimated directly by calculating the fraction of UMIs of alt-allele. When the reads were extracted from the bam files, including both WGS data and scRNA-seq data, only the ones with a Phred score larger than 30 at the given SNV position were kept for further calculation.

### Dimension Reduction and T-Distribution Stochastic Neighbor Embedding Projection

Dimension reduction was performed by principal component analysis (PCA) and visualized by t-distribution stochastic neighbor embedding (tSNE), following our previous study (Qin et al., 2021). The cells were colored accordingly to the inferred cell types, sample stages, or cell states. When displaying the expression pattern, highlighted cells were colored according to the allele expression, with their size scaled to log_10_ of the UMI counts.

### Identification of Monoallelic Expressions

Cells from each sample, each cell type, or each subpopulation (e.g., Norm) were pooled together to detect the constitutive MAEs, in a way that the common concerns for the scRNA-seq data, e.g., allelic drop-outs (ADOs), noise, and sparseness, were largely alleviated or canceled out (Borel et al., 2015; Castel et al., 2015). To increase the statistical power and reduce the false positives, SNVs observed in at least 10 cells were used for further analyses. We first identified the SNVs showing significantly biased allele expression against the expected balanced expression (by *χ*^2^-test). We further defined the SNVs showing serious deviation, in which UMI fractions of the minor allele were <5%, as constitutive MAE while other SNVs showing mild biased allelic expression were defined as allelic imbalanced expression (AIM). The constitutive MAEs in BMMCs were excluded from the cell-type-specific MAEs.

To detect random MAE (rMAE) at single-cell level, we only consider the SNV supported by >5 UMIs in a cell (i.e., “qualified” cell), thus the observed MAE of a SNV was not caused by chance, under an assumption of the binomial process (*p* < 0.05). This criterion leads to exclusion of a lot of SNVs and cells, leaving the SNVs possibly representing moderately and highly expressed genes, which are less affected by the technical variations (Deng et al., 2014; Kim et al., 2015; Zhao et al., 2017; Fan et al., 2018; Stamoulis et al., 2019) and undergoing relative fast transcriptional bursting (Kim and Marioni, 2013; Reinius and Sandberg, 2015; Stamoulis et al., 2019). A SNV was identified as single-cell MAE if its UMI of the minor allele was less than 1 or less than 5% of its total UMI counts of the two alleles, following the previous study (Reinius et al., 2016). The rMAE was defined as MAE excluding the constitutive MAEs and cell type specific MAEs. The fraction of rMAEs per cell was calculated by rMAE number dividing by the number of SNVs passed the “5-UMI” criterion. The cell fraction of a rMAE was measured by the proportion of the cells that monoallelically expressed the certain allele among the qualified cells.

### Permutation of Random MAEs

To address the contribution of randomness in the observed rMAE, we permuted the observed alleles of each SNV across observed cells to calculate the expected proportion of single-cell rMAEs. More specifically, for each SNV, we pooled the allele UMIs across the observed cells together, from which allele UMIs were sampled into each cell according to its original count. Then, we used the same criterion to identify the expected rMAEs in cells. The same procedure was used to test the significance of biallelic mosaic rMAEs in balanced expressed SNVs, the two alleles of which were not significantly biased in pooled cells (*p* > 0.05; *χ*^2^-test). All the permutations in the analysis were done by 1,000 times.

### Detection of Leukemia-Specific Monoallelic Expressions

Pairwise comparisons were conducted to detect the leukemia-specific MAEs among three cell subpopulations (i.e., Norm, preR.Leuk, and postR.Leuk). For each pair (e.g., preR.Leuk comparing with Norm), we first selected the MAEs only in the test cells (e.g., preR.Leuk), and then tested if two alleles of each MAE were expressed with significant difference between the two cell subpopulations, by Fisher’s exact test (*p* < 0.05). For detection of the leukemia-differentiated rMAEs in single cells, we only included the rMAEs that were shared between the comparing pairs. Cell numbers of the rMAEs and non-MAEs in each cell subpopulation were pair-wise compared by Fisher’s exact test (*p* < 0.05).

### Annotation and Enrichment Analysis

The SNVs were annotated by ANNOVAR (Wang et al., 2010) with relevant databases and assigned to genes according to their locations within the gene region. The gene enrichment analyses were performed by Metascape with default parameters and background gene set (Zhou et al., 2019)^1^. For cell type specific MAEs, the genes that were expressed in cells of the corresponding cell type were chosen as the background gene set, e.g., B cells.

### Statistical Analysis

All the statistical analyses in the study were conducted in R, and if not specified, the Fisher’s exact test was applied. When it was necessary, the BH method (Benjamini and Hochberg, 1995) was used for multiple test corrections.

### Data Availability Statement

Publicly available datasets were analyzed in this study. These data can be found here: https://ngdc.cncb.ac.cn/, *HRA000084* and *CRA000588*. The code used in this study has been deposited in https://github.com/faculty/MonoAlleleExpr.

## Results

### Identification of Constitutive Monoallelic Expression in Bone Marrow Mononuclear Cells

The BMMCs were obtained from a boy diagnosed with acute lymphoblastic leukemia (Qin et al., 2021). The BMMCs from the boy at complete remission are treated as normal BMMCs for analyzing MAE during hematopoiesis. After series of quality control, 7,016 cells were left for further analyses. The boy’s saliva sample was used for WGS (Zhang et al., 2018). SNVs were identified in WGS data using GTAK (McKenna et al., 2010; DePristo et al., 2011). We further filtered out SNVs by the following three conditions: (1) SNVs in CNV regions; (2) SNVs not in dbSNP database; and (3) SNVs detected in less than five cells. Finally, we obtained 83,174 SNVs for MAE analyses, with a median number of 287 SNVs per cell (Supplementary Table 1). For each SNV, the allele that is the same as the reference is called a ref-allele, while the other allele is called an alt-allele. The number of UMI was used to represent the expression level of each allele.

**FIGURE 1:**
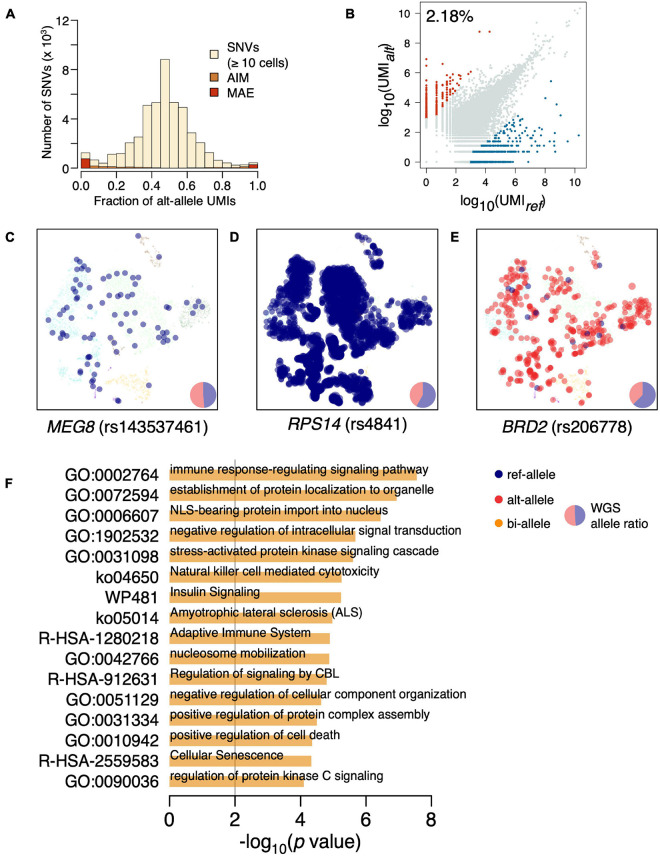
Identification of constitutive MAEs in BMMCs. **(A)** Histogram of VAF in BMMCs estimated by UMI counts. AIM expression and MAE are highlighted. **(B)** Identification of constitutive MAEs, with blue representing MAEs of reference allele while red representing MAEs of the alternative allele. The percentage of the constitutive MAE is shown on the top left of the plot. **(C)** tSNE projection of BMMCs (background), colored by expressed allele of *MEG8*. The size of the highlighted dot is scaled to log_10_ of UMI count. The pie chart in the bottom right shows the genetic allelic ratio of the two alleles from whole-genome sequencing (WGS) data. **(D)** tSNE projection of BMMCs, colored by expressed allele of *RPS14*. **(E)** tSNE projection of BMMCs, colored by expressed allele of *BRD2*. **(F)** GO enrichment analysis of the constitutive MAEs in BMMCs.

The distribution of variant allele frequency (VAF) estimated by UMI fraction across all cells was almost symmetrically centered in 0.5 (Figure 1A and Supplementary Figure 1). There are increased SNVs at both tails of the VAF distribution, suggesting the existence of biased allelic expression. We further separate the biased allelic expression into mildly biased allelic expressions [allelic imbalanced expression (AIM)] (*p* < 0.05; *χ*^2^-test) and strongly biased allelic expression with UMI fractions of the minor allele <5%. The strongly biased allelic expressions are constitutive MAEs across the BMMCs, accounting for 2.18% of the SNVs (Figure 1B and Supplementary Table 2). The constitutive MAEs contained several imprinted genes, such as *MEG8*, *NAP1L5*, and *IRAIN*; e.g., rs143537461 (C/A) located on imprinted gene *MEG8*, while only ref-allele (C) is exclusively expressed in BMMCs (Figure 1C). In addition to the imprinted genes, most of the detected constitutive MAEs are novel, indicating the existence of many hematopoiesis specific MAEs. For example, *RPS14*, showing strong constitutive MAE of reference allele (Figure 1D), is associated with hematopoiesis, particularly erythropoiesis (Wang et al., 2014; Schneider et al., 2016). *BRD2*, showing strong constitutive MAE of alterative allele (Figure 1E), is located in the MHC class II region and regulates the expression of many genes involved in immune pathways (Wang et al., 2021). GO enrichment analysis of constitutive MAE showed the immune relevant functional categories are significantly enriched (Figure 1F); e.g., “immune response-regulating signaling pathway” (*p* = 2.78e^–8^) and “adaptive immune system” (*p* = 1.27*e*^–5^).

### Constitutive Monoallelic Expressions in Major Cell Types of Bone Marrow Mononuclear Cells

**FIGURE 2:**
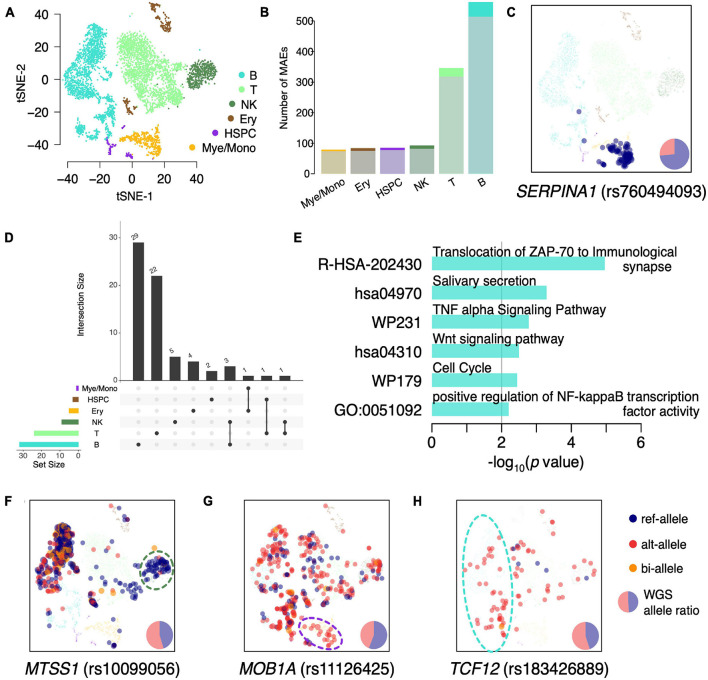
Identification of cell type specific MAEs in BMMCs. **(A)** tSNE projection of BMMCs, colored by inferred major cell types. **(B)** Detected number of MAEs in each major cell type. The shadowed (bottom) and bright (top) colors indicate the constitutive MAEs in BMMCs and cell type specific MAEs, respectively. **(C)** tSNE projection of BMMCs, colored by expressed allele of *SERPINA1*. **(D)** The upset plot of the cell type specific MAEs. **(E)** GO enrichment analysis of the B cell specific MAEs. **(F**–**H)** tSNE projection of BMMCs, colored by the expressed allele of *MTSS1* **(F)**, *MOB1A* **(G)**, and *TCF12* **(H)**.

We classified the BMMCs into six major cell types: T cells (38.33%; *CD3D*, *CD3E*, and *CD3G*), B cells (35.31%; *CD79A*, *CD79B*, and *CD19*), natural killer (NK) cells (9.21%; *FCGR3A* and *NCAM1*), myelocytes/monocytes (Mye/Mono; 8.55%; *LYZ*, *CD14*, and *CD68*), erythroid cells (Ery; 5.97%; *HBB* and *HBA2*), and hematopoietic stem and progenitor cells (HSPC; 2.64%; *CD34* and *AVP*) (Figure 2A). We then identified the constitutive MAEs in each of the six hematopoietic cell types. Interestingly, the majority of constitutive MAEs identified in each cell type were overlapped with that in BMMCs (Figure 2B and Supplementary Table 3), indicating MAEs are either conserved during development or highly shared between/among different cell types. These cell type shared constitutive MAEs include *HLA-DQB2* (B cells), *IL32* (T cells), and *SERPINA1* (Mye/Mono). For example, *SERPINA1*, identified as constitutive MAEs in BMMCs and only expressed in Mye/Mono (Figure 2C), participates in the monocyte recruitment and proinflammatory activation (Moraga et al., 2001; Janciauskiene et al., 2007). *IL32*, identified as constitutive MAEs in BMMCs, T cells, and NK cells (Supplementary Figure 2A), is a cytokine involved in inflammation and cancer development.

Monoallelic expressions that are identified in specific cell types but not in the BMMCs constitutive MAEs are called as cell type specific MAEs. There are only a few MAEs shared among these cell types (Figure 2D). GO enrichment analysis of B cell specific MAEs showed that they were enriched in the immune process including “TNF-α signaling pathway” (*p* = 1.66e^–3^) and “positive regulation of NF-κB transcription factor activity” (*p* = 6.25*e*^–3^) (Figure 2E). These cell type specific MAEs include *HLA-DRB5* (HSPC and Mye/Mono), *ZNF83* (Ery), *NUP210* (T cells), *MTSS1* (NK cells), *MOB1A* (Mye/Mono), and *RFTN1* and *TCF12* (B cells). For example, *MTSS1*, showing NKcell specific MAE (Figure 2F), is a tumor suppressor gene in leukemia (Yu et al., 2012; Schemionek et al., 2016) and plays an important role in the development of B cells (Yu et al., 2012). *MOB1A*, showing Mye/Mono cell specific MAE (Figure 2G), involves in the regulation of organ size and tumor growth by enhancing apoptosis. *TCF12*, showing B cell specific MAE (Figure 2H), is a transcription factor that regulates gene expression during hematopoiesis. *HLA-DRB5*, which plays an important role in antigen presentation, shows HSPCs and Mye/Mono cell specific MAE (Supplementary Figure 2B). *NUP210*, as a cell-intrinsic regulator of TCR signaling and T cell homeostasis (Borlido et al., 2018), shows T cell specific MAE (Supplementary Figure 2C).

### Identification of Random MAEs at Single-Cell Level

**FIGURE 3:**
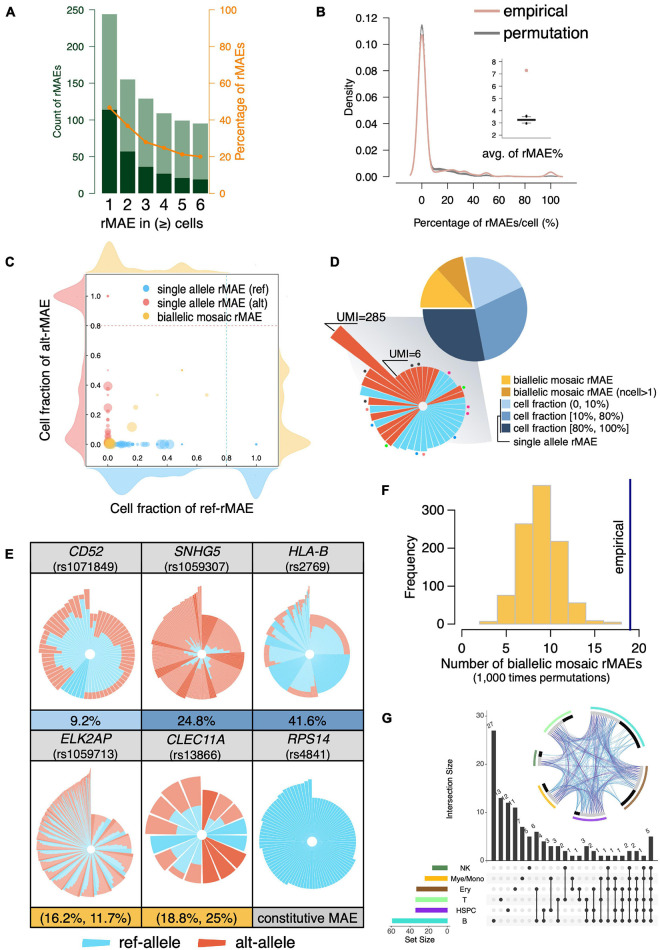
Identification of rMAEs and their features. **(A)** The identified rMAEs with different thresholds of the observed number of cells. The non-rMAEs and rMAEs are represented by olive and dark green, respectively. The orange line shows the percentage of rMAEs in the analyzed SNVs. **(B)** The percentage of rMAEs per cell (red), comparing with permutations of alleles (gray). The inset shows the average of the percentages of rMAEs per cell. **(C)** The rMAEs, including single-allele rMAE (red/blue) and biallelic mosaic rMAE (yellow), are detected in different fractions of cells. The density plots around the scatter plot show the density of rMAEs of different cell fractions with the corresponding allele. **(D)** The pie chart shows the fractions of different types of rMAEs. The single-allele rMAEs of high cell fraction are shown in circular stacked bars in the shadowed sector. Each bar represents a cell with stacked UMI counts (log_10_) of ref-allele (blue) and alt-allele (red) of a rMAE. The dots with the same color indicate the bars (cells) of the same rMAE, while bars without a dot mean that they are of different rMAEs. **(E)** Circular stacked profile of several rMAEs at the single-cell level. Each bar represents a cell with stacked UMI counts (log_10_) of ref-allele (blue) and alt-allele (red). Cells are ordered by the total UMI counts and the fraction of the ref-allele. Highlighted bars are cells showing MAE. The fraction of cells showing MAE is shown under the circular stacked profile. A constitutive MAE (*RPS14*) is also displayed as a control. **(F)** The observed biallelic mosaic rMAEs (vertical line) are significantly more than that by allelic permutation (*p* < 0.001). **(G)** Sharing of the rMAEs among different cell types. The circos plot shows the shared genes (purple line) and pathways (blue line) among different cell types. Gray bar indicates the genes that are shared by other cell types, and black bar indicates the genes that are unique to the corresponding cell type.

The scRNA-seq is a powerful approach to systematically analyze rMAEs in BMMCs. After strict quality control, we identified 114 rMAEs in BMMCs at single-cell level, accounting for 20–40% of the highly expressed genes (Figure 3A), giving rise to 7.29% SNVs showed rMAE per cell (Figure 3B), which is a little lower than other studies (Deng et al., 2014; Reinius et al., 2016; Savova et al., 2016), possibly due to our strict criteria (see “MATERIALS AND METHODS”). It is interesting to examine to which extend the observed rMAEs could be explained “by chance”. We permuted (1,000 times) the alleles of each SNV by sampling from the pooled UMIs across all cells, which resulted in 3.25% SNVs showing rMAE per cell on average (Figure 3B). Therefore, more than half (55.39%) of the rMAEs in real data were not observed by chance.

The rMAEs were further divided into single-allele rMAE and biallelic mosaic rMAE, with percentages of 78.1% and 21.9%, respectively (Figures 3C,D). The fractions of cells showing rMAEs vary a lot among different single-allele rMAEs (Figures 3C,D). Further investigation showed that most of the rMAEs of high cell fractions, which accounted for 36.0% of the single-allele rMAEs, were observed in only one qualified cell, but with UMI counts ranging from 6 to as high as 285 (Figure 3D). The single-allele rMAEs include *CD52* (rs1071849), *SNHG5* (rs1059307), and *HLA-B* (rs2769), e.g., 9.2% of the cells show rMAE on *CD52* (Figure 3E). For biallelic mosaic rMAE, the fraction of the cells showing rMAE is low or intermediate thus has not been detected in constitutive MAEs (Figures 3C,E). For example, the fractions of cells showing ref-allele rMAE and alt-allele rMAE at *ELK2AP* (rs1059713) are 16.2% and 11.7%, respectively (Figure 3E). The fractions of cells showing ref-allele rMAE and alt-allele rMAE at *CLEC11A* (rs13866) are 18.8% and 25%, respectively (Figure 3E). Furthermore, we permutated alleles of the biallelic mosaic rMAE and got rMAEs ranging from 2 to 18, which is significantly less than that of empirical value (*n* = 19) (*p* < 0.001; permutation test) (Figure 3F), indicating that the biallelic mosaic rMAEs were not observed by chance.

We then interrogated the rMAE by the cell type. As the largest cell group, we detected 59 rMAEs in B cells, while there were 34 rMAEs in the smallest cell group (HSPCs), which was the same as that in T cells (*n* = 34). Among the 114 rMAEs, about one-third (*n* = 39) was shared by at least two cell types (Figure 3G). Comparing with constitutive MAEs, the rMAEs are more shared between cell types, indicating they are less cell type specific or the stochasticity to increase the cell heterogeneity, despite that they represent a range of highly expressed genes.

### Leukemia Cells Showing Increased Constitutive Monoallelic Expressions and Random MAEs

**FIGURE 4:**
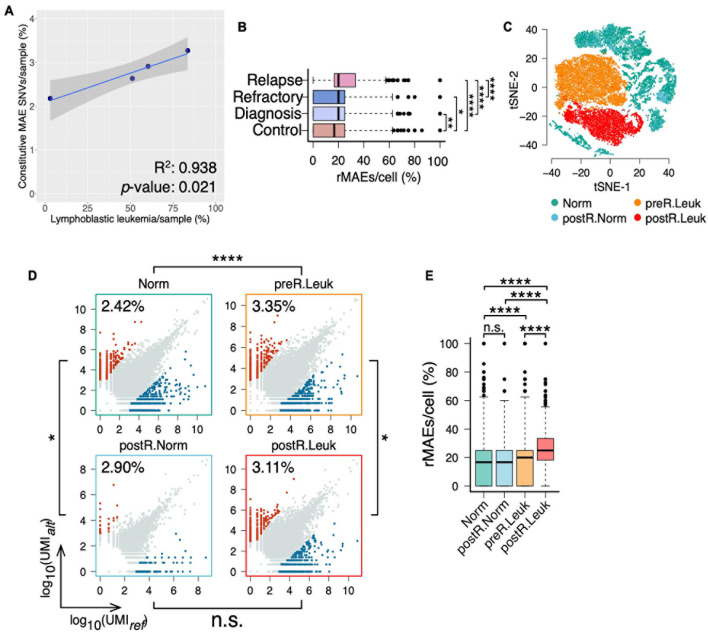
Leukemia cells showing increased constitutive MAEs and rMAEs. **(A)** The fractions of constitutive MAEs are correlated with the fractions of leukemia cells in BMMCs. **(B)** The fractions of rMAE per cell in BMMCs from control, diagnosis, refractory, and relapse. **(C)** tSNE projection of all BMMCs from the boy diagnosis with leukemia, colored by the inferred cell states, namely, leukemia cells before relapse (preR.Leuk), non-leukemia cells before relapse (Norm), leukemia cells after relapse (postR.Leuk), and non-leukemia cells after relapse (postR.Norm). **(D)** Constitutive MAEs of Norm cells, preR.Leuk cells, postR.Norm cells, and postR.Leuk cells. **(E)** The fractions of rMAE per cell of Norm cells, preR.Leuk cells, postR.Norm cells, and postR.Leuk cells.*p < 0.05, **p < 0.01, ***p < 0.005, and ****p < 0.001.

In addition to analyzing “normal” BMMCs, the BMMCs at diagnosis, refractory, and relapse of the same boy were analyzed for studying the changes of MAEs in leukemia (Supplementary Table 1). Interestingly, analysis of the four samples showed that fractions of leukemia cells were correlated with the fractions of constitutive MAEs (Supplementary Figure 3 and Figure 4A). We further identified the rMAEs in each cell of the four samples and found that “normal” BMMCs showed the lowest fraction of rMAEs and BMMCs at relapse showed the highest fraction of rMAEs, while the other two leukemia samples showed intermediate values (Figure 4B). Analyses of constitutive MAEs and rMAEs manifested that leukemia samples showed increased MAEs, thus we expected much stronger MAEs in leukemia cells since the normal cells in leukemia samples may not contribute to the increased MAEs.

After PCA, we clustered these BMMCs into normal cells, leukemia cells before relapse (preR.Leuk), and leukemia cells after relapse (postR.Leuk). Normal cells were further separated into normal cells before relapse (Norm) and normal cells after relapse (postR.Norm) (Supplementary Figure 4 and Figure 4C). There are 2.41% and 2.90% SNVs showing constitutive MAE in Norm and postR.Norm, respectively, while 3.35% and 3.11% in preR.Leuk and postR.Leuk, respectively (Figure 4D), thus leukemia cells have increased constitutive MAEs comparing with normal cells. The rMAE per cell between Norm and postR.Norm is not significantly different (Figure 4E). The leukemia cells from both preR.Leuk and postR.Leuk showed significantly higher levels of rMAE per cell, with postR.Leuk showing the highest value (Figure 4E). The results showed that separating the leukemia cells from normal cells in the leukemia samples made their difference more pronounced.

### Analyzing the Leukemia Cell-Specific Monoallelic Expressions

**FIGURE 5:**
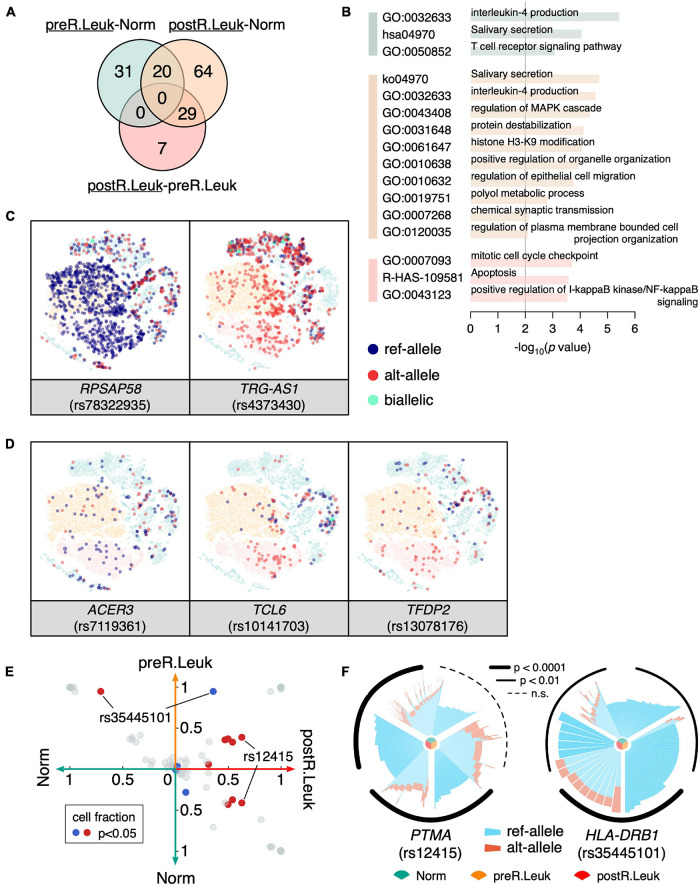
Analyzing the leukemia cell-specific MAEs. **(A)** Venn diagram shows constitutive MAEs specific to preR.Leuk and postR.Leuk (underlined), after pairwise comparisons. The green circle indicates preR.Leuk-specific MAEs, comparing with Norm, the orange circle indicates postR.Leuk-specific MAEs, comparing with Norm, while the red circle indicates postR.Leuk-specific MAEs, comparing with preR.Leuk. **(B)** GO enrichment analysis of the leukemia cell-specific MAEs. The colors match with that in **(A)**. **(C)** *RPSAP58* and *TRG-AS1* show Leukemia specific MAEs. **(D)** *ACER3*, *TCL6*, and *TFDP2* show postR.Leuk-specific MAEs. **(E)** Pairwise comparison of the detected rMAEs among Norm, preR.Leuk, and postR.Leuk. Each dot represents a rMAE, and the axis indicates the cell fraction of each rMAE, within the corresponding cell sub-population. Significantly biased (*p* < 0.05) rMAE in terms of the cell fraction is highlighted in red/blue. **(F)** Significantly differentiated rMAE among Norm cells, preR.Leuk cells, and postR.Leuk cells. An increased number of cells showed MAE of ref-allele on *PTMA* (rs12415) in postR.Leuk. *HLA-DRB1* (rs35445101) shows increased MAE in preR.Leuk but deceased in postR.Leuk.

Since we found that leukemia cells showed increased MAE, it would be more interesting to identify the leukemia cell-specific MAEs that potentially play an important role in leukemogenesis and leukemia development. Although leukemia cells showed increased MAEs comparing with normal cells, we only detected a few constitutive MAEs showed significant differences between normal cells and leukemia cells (Figure 5A), which indicated that most of the MAE changes between normal cells and leukemia cells are weak. GO enrichment analysis showed that immune-associated categories were commonly shared by the differentiated MAEs among Norm, preR.Leuk, and postR.Leuk, e.g., “IL-4 production” and “positive regulation of I-κB kinase/NF-κB signaling”. The postR.Leuk-specific MAEs were enriched in “histone H3-K9 modification” and “mitotic cell cycle checkpoint” and “apoptosis”, comparing with Norm and preR.Leuk, respectively (Figure 5B). Among these leukemia cell-specific MAEs, *RPSAP58* (rs78322935) and *TRG-AS1* (rs4373430) only expressed one allele in leukemia cells (Figure 5C). *TRG-AS1* is a lncRNA and regulates cancer progression by interacting with other microRNAs (Xie et al., 2019; He et al., 2020; Sun et al., 2020). We further identified the relapse-specific constitutive MAEs, which include *ACER3*, *TCL6*, and *TFDP2* (Figure 5D). *ACER3* coregulates cell proliferation and survival with *ACER2* (Hu et al., 2010) and plays an important role in leukemia development (Chen et al., 2016); while *TCL6* is associated with clinical outcomes of B-cell acute lymphoblastic leukemia patients (Cuadros et al., 2019); *TFDP2* plays core roles in apoptosis and cell proliferation (Korz et al., 2002). Altogether, most of the significantly changed MAEs were involved in immune pathways and regulation of cell proliferation, thus could explain the association between increased MAEs and the dysfunction in leukemia cells.

We also identified the significantly different rMAEs among Norm, preR.Leuk, and postR.Leuk (Figure 5E). Among these different rMAEs, 62.8% postR.Leuk showed rMAE at *PTMA* (rs12415), which is significantly higher than that of Norm (∼41.4%) and preR.Leuk (∼38.8%) (Figure 5F). Notably, *PTMA* is associated with lymphocyte proliferation and apoptosis in leukemia (Gómez-Márquez et al., 1989; Fan et al., 2006), thus finding the change of rMAE on *PTMA* has a lot of implications. The *HLA-DRB1* (rs35445101) shows high reference allele rMAE in Norm (∼70.8%) and preR.Leuk (∼94.7%), while postR.Leuk maintains the lowest reference allele rMAE (35.7%) among these cell populations (Figure 5F). *HLA-DRB1* plays a central role in antigen presentation and the decreased reference allele rMAE may impact its function.

## Discussion

Mammalian genomes are diploid, we usually just assume both alleles are equally expressed and did not consider the differences between the bialleles (Jin et al., 2012; Han et al., 2020). In this way, most studies only analyzed the average gene expression profile of the two alleles, even though MAE has been discovered during analyses of X-chromosome inactivation in the 1960s (Lyon, 1986), partially because most people do not realize the prevalence of MAE. Large-scale interrogations of MAEs have demonstrated MAEs were widespread in mammalian cells (Gimelbrant et al., 2007; Zwemer et al., 2012; Deng et al., 2014; Gendrel et al., 2014; Savova et al., 2016). The advance of scRNA-seq provides new biological insight on MAE, although most studies only used hundreds of cells (Deng et al., 2014; Borel et al., 2015; Kim et al., 2015; Reinius et al., 2016). Taking advantage of high throughput scRNA-seq with about 31,000 single-cell transcriptomes from the same individual, this study provides a fine scale landscape of MAE in hematopoiesis, at sample level, cell type level, and single-cell level. In addition to the known imprinted genes, we detected a lot of novel MAEs in BMMCs. As a cross validation, we found more than three quarters of the constitutive MAEs were reproducible in the bulk RNA sequencing of the same individual. The MAEs are associated with immune functions, which may indicate that the diversity of immunity is attributed to MAE.

We detected a considerable number of rMAE at single-cell level. Interestingly, a cell can stochastically express either of the two alleles thus leading to different cells expressing different alleles, which is called biallelic mosaic rMAEs. With a small but significant number, such genes are presumably increasing the cellular heterogeneity when the two alleles are different. Meanwhile, the biallelic mosaic rMAEs might be caused by transcriptional bursting thus it is the outcome of this important periodic switching kinetics. Furthermore, we observed much higher MAE levels in leukemia cells than that in normal cells, indicating the association between MAE and leukemogenesis. Leukemia-specific MAEs, including *TCL6*, *TFDP2*, and *PTMA*, are reported to be associated with tumorigenesis and cell proliferation. It is interesting to detect the *TCL6* in leukemia-specific MAEs, since it was recently reported that low *TCL6* levels were associated with poor survival of B-cell ALL patient, through a link between *TCL6*, *TCL1B*, and the *AKT1* pathway (Cuadros et al., 2019). The monoallelic expression may be indicative of insufficient dosage or expression deficiency of *TCL6* in our sample, who was also a B-cell ALL patient. Another interesting gene would be *PTMA*, which shows significantly higher proportion of MAE cells in the relapsed sample, and studies demonstrated that, though in other types of cancers, it can predict recurrence and poor prognosis (Ha et al., 2015; Chen et al., 2018). The observation that a higher level of MAE was in line with altered epigenetic regulations of leukemia (Miles et al., 2020; Waanders et al., 2020). MAE is also highly mediated by epigenetics, such as DNA methylation and histone modifications (Eckersley-Maslin and Spector, 2014a; Reinius and Sandberg, 2015), and interestingly, we found an enrichment for “histone H3-H9 modification” in relapsed leukemia cells (Figure 5B).

In summary, as far as we know, this is the first systematic study on MAEs in human BMMCs using scRNA-seq and analyzed MAE in three layers including sample level, cell type level, and single-cell level. We found increased MAEs (both constitutive and random) in leukemia cells by comparing with normal cells, indicating the association between MAE and leukemogenesis. Particularly, these leukemia-associated MAEs may be the epigenetically therapeutic targets of leukemia.

## Data Availability Statement

Publicly available datasets were analyzed in this study. This data can be found here: https://ngdc.cncb.ac.cn/, HRA000084 and CRA000588.

## Author Contributions

RF conducted major work of data analysis and interpretation. PQ performed process of the scRNA-seq data. NH collected and integrated the data. WJ conceived and supervised theproject. YW interpreted the results and co-supervised the project. ZH and XZ coordinated the cooperation. WJ, RF, and YW drafted the manuscript, with critical revisions by NH, ZH, and XZ. All authors contributed to manuscript revision, read and approved the final manuscript for publication.

## Conflict of Interest

The authors declare that the research was conducted in the absence of any commercial or financial relationships that could be construed as a potential conflict of interest.

## Publisher’s Note

All claims expressed in this article are solely those of the authors and do not necessarily represent those of their affiliated organizations, or those of the publisher, the editors and the reviewers. Any product that may be evaluated in this article, or claim that may be made by its manufacturer, is not guaranteed or endorsed by the publisher.

## Footnotes

1
https://metascape.org

## Supplementary Material

Supplementary Figure 1 Q-Q plot of VAF of in BMMCs, estimated by UMI counts.

Supplementary Figure 2 tSNE projection of BMMCs (background), colored by the expressed allele of *IL32* **(A)**, *HLA-DRB5* **(B)**, and *NUP210* **(C)**. The size of the highlighted dot is scaled to log_10_ of its UMI count, and the pie chart in the bottom right shows the genetic allelic ratio of the two alleles from WGS data.

Supplementary Figure 3 Identification of constitutive MAEs in diagnosis **(A)**, refractory **(B)**, and relapse **(C)**, with blue representing MAE of the reference allele while red representing MAE of the alternative allele. The percentage of the SNVs with constitutive MAE is shown on the top left of the plot.

Supplementary Figure 4 tSNE projection of all BMMCs from the boy diagnosed with leukemia, colored by sampling times, namely control, diagnosis, refractory, and relapse.
