# Supplementary figures and images for: A Comprehensive Characterization of Monoallelic Expression During Hematopoiesis and Leukemogenesis via Single-Cell RNA-Sequencing
Source: Front Cell Dev Biol. 2021 Oct 13;9:702897. doi: 10.3389/fcell.2021.702897 (PMC8548578; doi:10.3389/fcell.2021.702897)

# Normal Q-Q Plot

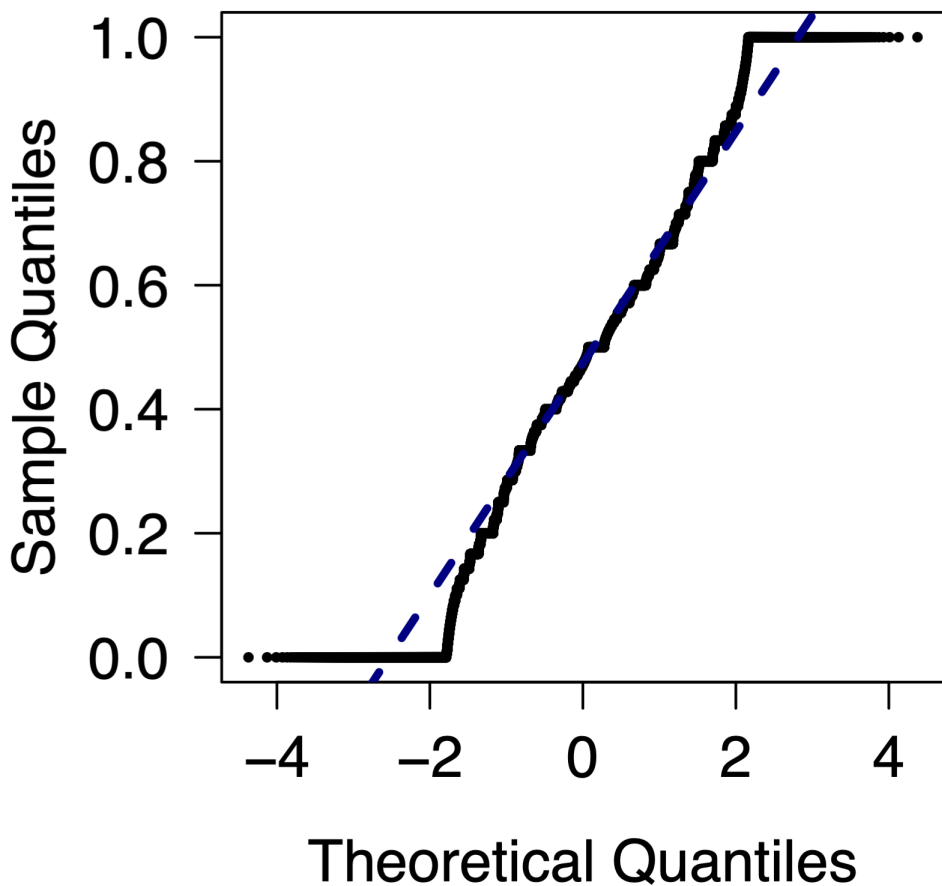

(A)

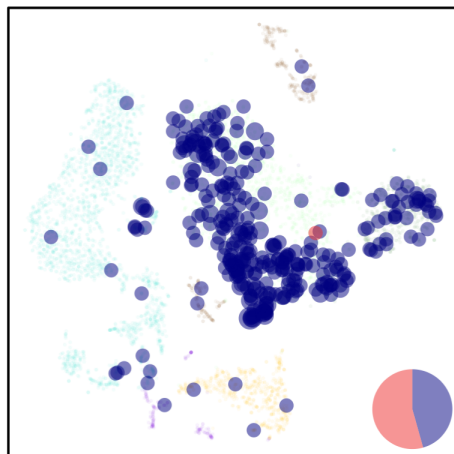

*IL32* (rs34184287)

● ref-allele  
● alt-allele  
● bi-allele

WGS  
allele ratio

(B)

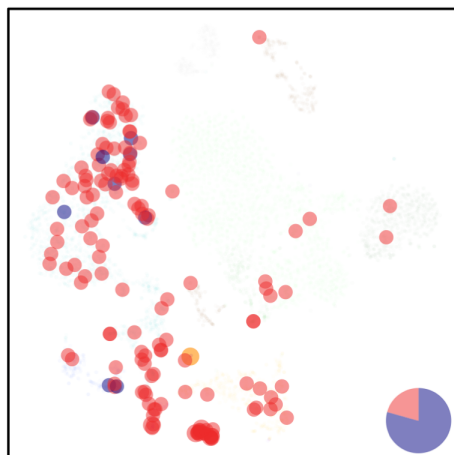

*HLA-DRB5* (rs1141867)

(C)

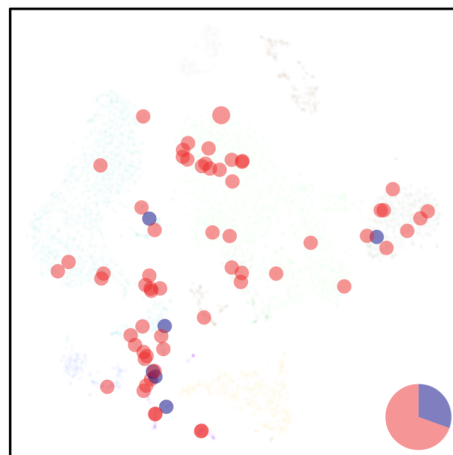

*NUP210* (rs9884019)

**(A)**

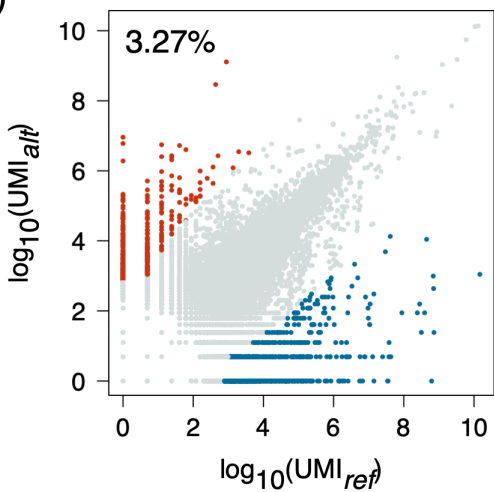

**(B)**

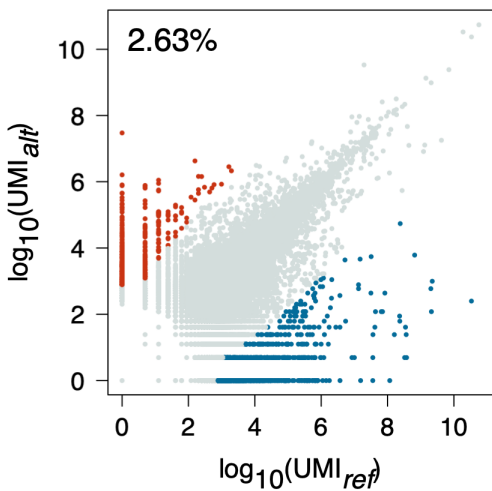

**(C)**

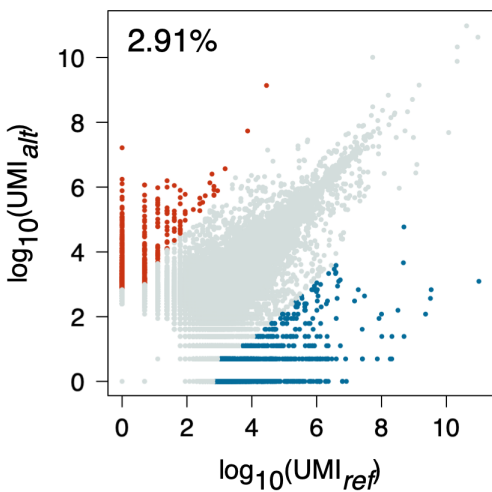

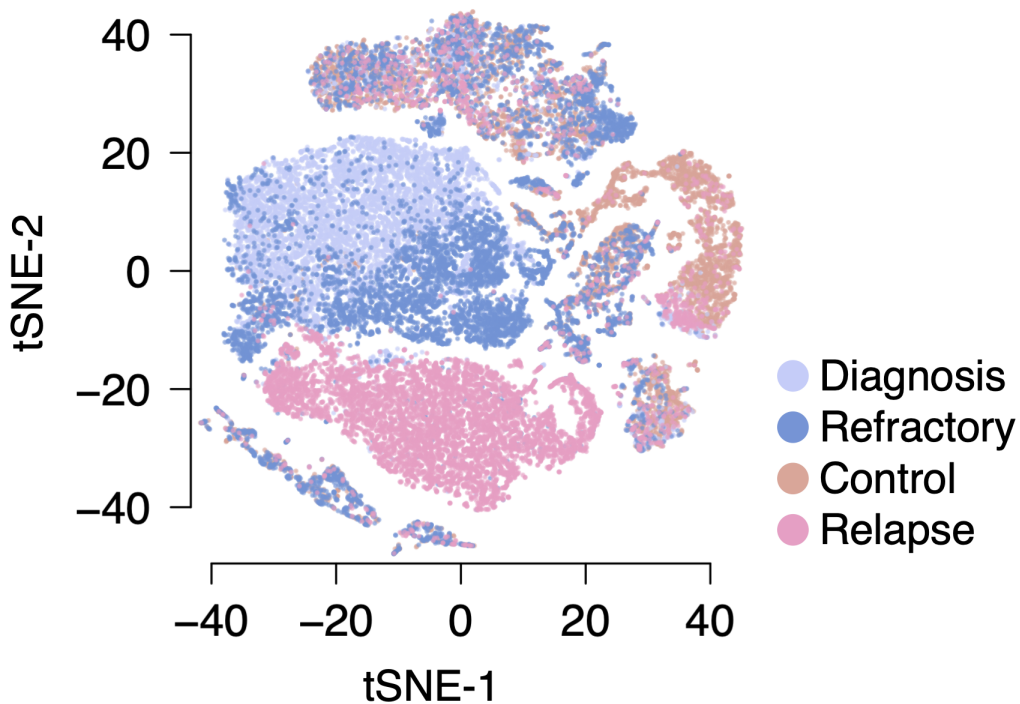

Supplement: Supplementary Figure 1 — Q-Q plot of VAF of in BMMCs, estimated by UMI counts. [file Data_Sheet_1.PDF]
